# miRNAs and resistance to EGFR—TKIs in *EGFR*-mutant non-small cell lung cancer: beyond ‘traditional mechanisms’ of resistance

**DOI:** 10.3332/ecancer.2015.569

**Published:** 2015-09-02

**Authors:** Biagio Ricciuti, Carmen Mecca, Matteo Cenci, Giulia Costanza Leonardi, Lorenzo Perrone, Clelia Mencaroni, Lucio Crinò, Francesco Grignani, Sara Baglivo, Rita Chiari, Angelo Sidoni, Luca Paglialunga, Maria Francesca Currà, Emanuele Murano, Vincenzo Minotti, Giulio Metro

**Affiliations:** 1 Medical Oncology, Santa Maria della Misericordia Hospital, Azienda Ospedaliera di Perugia, Perugia 06156, Italy; 2 Department of Experimental Medicine, University of Perugia, Perugia 06156, Italy; 3 Department of Clinical and Experimental Medicine, Division of Pathology, University of Perugia, Perugia 06156, Italy

**Keywords:** EGFR mutation, EGFR-TKI, NSCLC, miRNAs, resistance

## Abstract

Epidermal growth factor receptor (EGFR)-tyrosine kinase inhibitors (TKIs) have dramatically changed the prognosis of advanced non-small cell lung cancers (NSCLCs) that harbour specific *EGFR* activating mutations. However, the efficacy of an EGFR-TKI is limited by the onset of acquired resistance, usually within one year, in virtually all treated patients. Moreover, a small percentage of *EGFR*-mutant NSCLCs do not respond to an EGFR-TKI, thus displaying primary resistance. At the present time, several mechanisms of either primary and acquired resistance have been elucidated, and new drugs are currently under preclinical and clinical development in order to overcome resistance to treatment. Nevertheless, there still remains much to be thoroughly investigated, as so far research has mainly focused on the role of proteincoding genes involved in resistance to EGFR-TKIs. On the other hand, in line with the data underscoring the relevance of non-coding RNAs in the pathogenesis of lung cancer and modulation of response to systemic therapies, microRNAs (miRNAs) have been supposed to play an important role in resistance to EGFR-TKIs. The aim of this review is to briefly summarise the existing relationship between miRNAs and resistance to EGFR-TKIs, and also focusing on the possible clinical applications of miRNAs in reverting and overcoming such resistance.

## Introduction

Lung cancer is the leading cause of cancer-related deaths worldwide, representing 27.2% of all cancer deaths [1]. As many other neoplasms, lung cancer has been recognised as a group of different diseases, each with its own clinical and biological features. On one hand, non-small cell lung cancer (NSCLC), which accounts for approximately 80% of all lung cancers, on the other, small cell lung cancer (SCLC), which accounts for the rest of cases [2]. Even though enormous improvements have been made in the systemic treatment of advanced NSCLC, particularly for the non-squamous subgroup, the prognosis of these patients is still extremely poor, five-year survival being 5% and 1%, respectively [3]. The reason for this poor outcome should be attributed, at least in part, to the fact that platinum-based chemotherapy represents the standard of care for the majority of patients with advanced disease, irrespective of tumour biology, which does not take into account the intra- and inter-individual heterogeneity of each lung cancer case [4]. Nevertheless, recent breakthroughs in the understanding of NSCLC biology have led to the discovery of specific genetic alterations, such as *EGFR* mutations, anaplastic lymphoma kinase (*ALK*), and *ROS1* gene rearrangements, each of which identifies a distinct disease entity which has been termed ‘oncogene addicted’ in order to reflect its dependence on a single genetic ‘driver’ for proliferation and survival of cancer cells.

With regard to *EGFR*-mutant NSCLC, the introduction for therapeutic use of EGFR-TKIs, either reversible, first-generation (gefitinib, erlotinib) or irreversible, second-generation (afatinib) agents, has dramatically improved the prognosis of patients whose tumour harbours this specific genetic alteration (Table 1) [5].

In this paper, we will briefly summarise the most well known mechanisms of resistance to EGFR-TKIs. Also, our exploration will go beyond traditional mechanisms of resistance, particularly focusing on the ability of microRNAs (miRNAs) to regulate response to EGFR-TKIs. Finally, the possible clinical applications of miRNAs in escaping resistance to EGFR-TKIs will be discussed.

## *EGFR* mutations and EGFR-TKIs in NSCLC

EGFR (ERBB1/HER1) is a member of a transmembrane receptors tyrosine kinase (TK) superfamily, which also includes ERBB2/HER2, ERBB3/HER3, and ERBB4/HER4 [19]. Similarly to other members, EGFR is characterised by an extracellular ligand-binding region, a single transmembrane region, and an intracytoplasmic region with TK activity [20]. When an activating somatic mutation occurs in the EGFR-TK domain, EGFR undergoes ligand-independent homo/hetero-dimerisation with another receptor of the same family. This leads to conformational changes in the three-dimensional structure of EGFR that promote ATP-mediated autophosphorylation of the TK domain, and subsequent receptor activation [21, 22]. As a consequence, multiple intracellular signalling cascades are implemented, such as RAS/RAF/MEK/ERK, JAK/STAT, and PI3K/AKT/mTOR, all of which result into intensification of pro-survival and anti-apoptotic signalling [19, 22].

Interestingly, although *EGFR* mutations are more commonly associated with specific clinicopathological features such as female gender, Asian ethnicity (where they can be found in up to 30% of advanced NSCLCs as opposed to 15% for the western population), non-smoking history, and adenocarcinoma histology [23], it is not possible to rule out the possibility of an *EGFR* mutation solely on the basis of the aforementioned characteristics. Also, only activating mutations that affect the TK domain (exons 18→21) are those who predict exquisite sensitivity to treatment with an EGFR-TKI, since EGFR-TKIs and ATP compete for binding to the same pocket on the EGFR-TK domain [24].

*EGFR* activating mutations include in frame deletions, in frame duplications/insertions, and point mutations [Murray 2008]. Specifically, the most represented and better characterised are the so called classic mutations, which include in frame deletions in exon 19 in correspondence of the LeuArgGluAla sequence (E746-A750), and the exon 21 point mutation Leu858Arg (L858R), together representing 85%–90% of all *EGFR* mutations in NSCLC [25]. On the other hand, a number of ‘uncommon’ mutations has been reported, such as G719X in exon 18 (G719C, G719S, G719A), L861Q in exon 21, and S768I in exon 20, whose predictive role to treatment with an EGFR-TKI is much less defined. Nevertheless, a growing body of evidence seems to confirm a positive predictive role of these uncommon mutations to treatment with an EGFR-TKI [26–28], although to a less extent than classic mutations [29]. Conversely, exon 20 in frame insertion as well as a *de novo* T790M point mutation in exon 20 are associated with primary resistance to EGFR-TKIs [26–28].

Recently, six phase III randomised trials comparing either a reversible (gefitinib or erlotinib) or irreversible (afatinib) EGFR-TKI versus platinum-based chemotherapy in patients with untreated *EGFR*-mutant advanced NSCLC showed superiority for the EGFR-TKI in terms of response rate (RR) and progression free survival (PFS) (Table 1) [6–18], as well as quality of life (QOL) [6, 13, 30]. These results corroborated further those of the earlier IPASS and first-SIGNAL trials, in which gefitinib was compared head-to-head with platinum-based chemotherapy in a population enriched for the presence of an *EGFR* mutation, such as East Asian and never/light smoker patients (Table 1) [6, 8]. In both studies gefitinib demonstrated a significant increase in RR and PFS only for the *EGFR*-mutant subgroup, whereas *EGFR* wild type patients were found to benefit more from standard chemotherapy than gefitinib. Importantly, although first-line afatinib significantly improved OS compared to chemotherapy in patients with EGFR Del19 mutations in two randomised trials, LUX-Lung 3 and LUX-Lung 6, there was no significant difference in OS of patients with EGFR L858R mutation treated with afatinib compared to chemotherapy, both individually and in pooled data [31]. These findings suggest that patients harbouring EGFR Del19 and L858R mutations may represent two separate populations and further studies are required to address this issue.

Unfortunately, despite the impressive activity of EGFR-TKIs in *EGFR*-mutant NSCLCs, virtually all patients would undergo resistance to treatment, usually within one year of treatment initiation. Against this background, it is of primary importance to understand the molecular mechanisms underlying resistance to EGFR-TKIs in order to develop novel therapeutic strategies aimed at improving further the prognosis of *EGFR*-mutant NSCLCs.

## ‘Traditional mechanisms’ of primary and acquired resistance to EGFR-TKIs

Currently, 20–30% of *EGFR*-mutant NSCLC patients do not undergo tumour shrinkage on treatment with an EGFR-TKI, thus identifying a primary resistant cohort. Furthermore, virtually all patients who have experienced either an objective response or prolonged disease stabilisation (≥ six months) on an EGFR-TKI will eventually develop resistance to the drug, which identifies the acquired resistant cohort [32].

With regard to primary resistance, exon 20 insertions have been invariably associated with lack of response to EGFR-TKIs, as suggested by numerous clinical studies [26–28, 33]. Also, *de novo* T790M point mutation in exon 20 identifies a group of patients who perform poorly on EGFR-TKIs. Although considered rather uncommon, highly sensitive method based on laser microdissection and peptide nucleic acid (PNA)-clamping polymerase chain reaction (PCR) recently unveiled an unexpected prevalence of *de novo* T790M substitution in patients with primary resistance to EGFR TKIs (2%–9%) [34–35]. This mutation, which is actually the major determinant of acquired resistance, is worthy of note and will be handled further along. Moreover, various alterations in the EGFR pathway may affect patients with NSCLC, thus conditioning response to EGFR-TKIs. More in detail, loss of phosphatase and tensin homolog PTEN expression, which is an onco-suppressor gene involved in the inhibition of PI3K/AKT/mTOR pathway, as well as *PIK3CA* activating mutations have both been reported *in vitro* as inducers of primary resistance to gefitinib and erlotinib through the blockade of EGFR-TKI-induced apoptosis in *EGFR*-mutant cell lines [34, 39]. In addition, BIM deletion polymorphism has been found to induce resistance to EGFR-TKIs [40]. BIM is a member of the BCL-2 pro-apoptotic proteins family, which is involved in Bax/Bak-mediated cytochrome c release and apoptosis. Of note, the relevance of BIM levels in influencing clinical response to EGFR-TKIs is supported by the results of EURTAC study, which randomised *EGFR*-mutant patients to erlotinib or platinum-based chemotherapy [15]. Interestingly, the erlotinibtreated patients who had low/intermediate mRNA levels of BIM expression experienced a lower PFS compared with those who had high levels of BIM expression [41]. Conversely, no differences in PFS were observed in the chemotherapy arm according to mRNA levels of BIM.

Currently, approximately 60% of cases of acquired resistance appear to be associated with the presence of a secondary missense mutation termed T790M. Such a mutation is characterised by the replacement of a threonine with a methionine at codon 790 of exon 20 of the EGFR gene, in a site that affects the catalytic adenosine 5’ triphosphate (ATP) binding pocket of the EGFR-TK domain [42]. That is because T790M mutation restores the binding affinity between the EGFR-TK domain and ATP, thus identifying what has been called the ‘gatekeeper’ mutation [43]. Of note, Maheswaran *et al* have shown that in some *EGFR*-mutation positive NSCLCs who develop T790M-mediated acquired resistance, this mutation could actually be detected at baseline in minor clones with the use of highly sensitive techniques, thus becoming dominant during exposure to EGFR-TKIs as a result of selective pressure induced by treatment with an EGFR-TKI [44]. Nevertheless, a few other EGFR secondary point mutations have been involved in acquired resistance to an EGFR-TKI, namely D716Y (exon 19), L747S (exon 19), and T854A (exon 21) [45–47]. In addition, acquired resistance might be because of the presence of other mutant signalling proteins (e.g. *PI3CKA* mutation, *HER-2* amplification, *BRAF* mutation) as well as to the activation of EGFR signalling pathways via other aberrant molecules. Among the latter mechanism, the most relevant is certainly *MET* gene amplification, as it drives roughly 5–10% of cases of acquired resistance to EGFR-TKIs [42]. *MET* encodes for a transmembrane TK receptor implicated in acquired resistance through the activation of the PI3K/AKT/mTOR pathway via interaction with ERBB3 [48]. Not surprisingly, hepatocyte growth factor (HGF), which is the only known ligand of MET receptor, has also been implicated both in primary and acquired resistance to EGFR-TKIs [49–51]. Finally, in a minority of cases some other additional mechanism may lead to the development of acquired resistance such as phenotypic changes in the tumour. This can be because of SCLC transformation as well as to epithelial-to-mesenchymal transition (EMT) [52–53]. Of note, cells displaying SCLC features still harbour a drug-sensitive *EGFR* mutation. On the other hand, an increasing body of evidence describes the molecular machinery involved in EMT, which includes a variety of phenotypic and molecular changes (e.g. loss of tight junction and cell adhesion, induction of a mesenchymal phenotype) responsible for the increased aggressiveness and invasiveness of the tumour [54]. Cells with EMT are associated with loss of E-cadherin and β-catenin, though they gain the expression of vimentin and N-cadherin. At a molecular level, NOTCH-1 upregulation, transforming growth factor TGF-β overexpression, activation of MET and AXL pathways have been identified as major determinants of EMT-induced acquired resistance to EGFR-TKIs [55–58]. Even though *EGFR* mutations are largely homogeneous, mechanisms of resistance are more heterogeneous. Multiple mechanisms of resistance can be detected in a single tumour specimen, and different mechanisms of resistance can emerge from different tumour sites in the same patient, indicating the polyclonal nature of TKIs resistance. For instance, by examining various progressive lesions in an autopsy case from a patient with acquired resistance to EGFR TKI, Balak and colleagues revealed that the seven different sites all harboured the exon 19 deletion, conversely the T790M mutation was found in six out of the seven sites, but was not detected in the brain metastasis, despite the use of highly sensitive procedure [45]. In addition, a recent large series study designed to assess prospectively the frequency of various mechanism of resistance in 155 patients revealed that 98 had a second-site EGFR T790M mutations (63%) and four had small cell transformation, whereas MET and HER-2 amplification were reported in four patients and three patients respectively. Even though acquired mutations in PIK3CA, AKT1, BRAF, ERBB2, KRAS, MEK1, and NRAS were not detected, the coexistence of different mechanisms of acquired resistance was observed in 4% of patients [59].

## miRNAs and resistance to EGFR-TKIs

MiRNAs represent a class of 18–25 nucleotides in length, single stranded, endogenous and evolutionarily conserved small non-coding RNAs. They bind protein-coding mRNAs subsequently leading to mRNAs degradation, storage, and translational inhibition [60]. Although miRNAs account for barely 1%–2% of the human genome, they actually regulate and supervise the activity of roughly 50% of all protein-coding genes [61]. Currently, a number of miRNAs has been described which may have a specific role in lung cancer pathogenesis, biological and clinical disease behaviour as well as in modulating response to anticancer treatments, particularly EGFR-TKIs (Table 2) [60–63].

**Figure 1.**
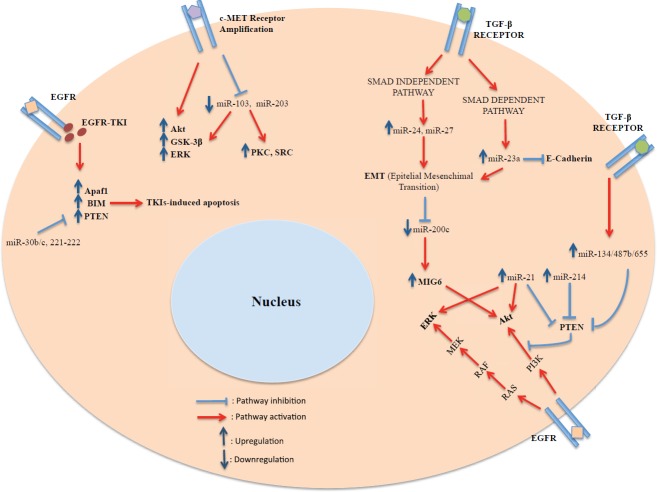
Schematic diagram of the relationship existing between miRNAs and resistance to EGFR-TKIs at a cellular level.

Located on chromosome 17q23.1, miR-21 is one of the most investigated miRNAs. Several studies reported its upregulation in NSCLC, and delineated its involvement in promoting lung cancer cell growth and invasion (Figure 1) [64–66]. Recently, Li *et al* demonstrated that miR-21 expression levels are higher in the *EGFR*-mutant (delE746-A750) and EGFR-TKI resistant NSCLC cell line PC9GR, inversely correlating with PTEN and PDCD4 expression [67]. On the other hand, miR-21 was significantly associated with the activation of the PI3K/Akt pathway. Notably, the same investigators found that miR-21 serum levels in *EGFR*-mutant NSCLC patients treated with an EGFR-TKI were significantly higher at the time of the onset of acquired resistance compared with baseline [67]. Additionally, in human *EGFR*-mutant NSCLC cell lines, miR-21 has been found to downregulate PTEN expression, a mechanism that could help promote lung carcinogenesis. MiR-21 expression has also been shown to associate with poor response and shorter overall survival (OS) in patients undergoing treatment with an EGFR-TKI [68]. Consistently, miR-21 overexpression decreased gefitinib sensitivity through PTEN downregulation and AKT/ERK pathway activation, whereas miR-21 knockdown restored gefitinib sensitivity through PTEN upregulation and AKT/ERK repression.

Similarly to miR-21, also miR-214 has been involved in acquired resistance to gefitinib through PTEN and PI3K/AKT pathway. More in details, Wang *et al* obtained gefitinib resistant cell line HCC827/GR through the exposure of normal HCC827 cells (NSCLC cell line with 746E-750A EGFR gene deletion) to increasingly larger concentrations of gefitinib and subsequently found that miR-214 was significantly overexpressed in HCC827/GR, also inversely correlating with the levels of PTEN expression [69]. Furthermore, not only miR-214 knockdown restored PTEN mRNA levels, but also was associated with p-Akt inactivation. Taken together, these data suggest that both miR-21 and miR-214 drive acquired resistance to gefitinib through the downregulation of PTEN expression, and therefore activation of the PI3K/AKT pathway independently of EGFR.

As previously mentioned, EMT represents an important determinant of clinical response to treatment with an EGFR-TKI, which has attracted much of an interest in miRNAs involved in the EMT phenomenon. Among them, miR-23a, miR-24, and miR-27a is a miRNAs cluster located on chromosome 19p13.12 with profound oncogenic properties in several human cancers. Cao *et al* showed that miR-23a is induced by the TGF-β1/Smad pathway in EGFR-WT A549 lung adenocarcinoma cells with the EMT phenomenon, whereas miR-24 and miR-27a are induced by a Smad-independent mechanism in the same cell lines [70]. These findings suggest that the activation of the TGF-β pathway contributes to EMT also through miR-23a/24/27a upregulation. In support of these data, there is evidence that miR-23a overexpression strengthens EMT and suppresses E-cadherin expression, whereas miR-23a knockdown restores E-cadherin expression [70]. Therefore, since miR-23a overexpression regulates TGF-β1-induced EMT and consequently EMT-related acquired resistance to gefitinib, miR-23a may be a new therapeutic target in both EGFR WT and *EGFR*-mutant NSCLC patients resistant to EGFR-TKIs.

Still on EMT, the miR-200 family has been found to be downregulated during TGF-β1-induced EMT, leading to mitogen-inducible gene 6 (MIG6) upregulation. MIG6 is a negative regulator of EGFR and its overexpression in TGF-β1-induced EMT changes the molecular profile of NSCLC cells to an AKT-activated/EGFR-independent state. Izumchenko *et al* showed that expression levels of MIG6 and miR-200 were significantly correlated with EMT, as well as resistance to erlotinib in 25 cancer cell lines originating from different tissues; also the MIG6 mRNA/miR-200 ratio was inversely correlated with response to erlotinib in EGFR wild-type NSCLC patients [71]. In another study, Kitamura *et al* identified three other miRNAs, namely miR-134, miR-487b, and miR-655, all induced after exposure to TGF-β1 in lung adenocarcinoma cells with EMT [72]. Notably, the upregulation of these mi-RNAs results in loss of PTEN function, as one of the predicted targets of this cluster of miRNAs is the protein MAGI2, which is an essential scaffold protein for the proper functioning of PTEN. Overall, miR-134/487b/655 cluster promotes TGF-β1-induced EMT phenomenon, thus conditioning the development of ETM-driven acquired resistance to EGFR-TKIs [72].

Other authors examined EGFR and MET mediated changes of miRNAs in NSCLC cell lines. More in details, they showed that gefitinib treatment reduces miR-30b/c and miR-221/222 expression levels, consequently leading to the upregulation of APAF and BIM, which are both involved in determining EGFR-TKIs-induced apoptosis in sensitive *EGFR*-mutant, HCC827, and PC9 NSCLC cell lines [73–74]. Conversely, miR-30b/c and miR-221/222 downregulation was not observed after gefitinib treatment in HCC827/GR and PC9GR resistant clones of gefitinib hypersensitive EGFR exon 19 mutant HCC827 and PC9 NSCLC cell lines [74]. The same authors also showed that MET activation reduces expression levels of miR-103 and miR-203, which normally function as oncosuppressor miRNAs through the inhibition of SRC and PKC activity. Additionally, miR-103 and miR-203 were found to be significantly upregulated in MET knockdown Calu-1 NSCLC cells, whereas their overexpression was strongly related to reduced phosphorylation of Akt, GSK3β, and ERKs. This might suggest that MET amplification and overexpression may drive acquired resistance to EGFR-TKIs by enhancing AKT/ERKs pathway even through miR-103 and miR-203 regulation [74]. Taken together, these studies indicate that miR-30b/c and miR-221/222 upregulation in NSCLC determine resistance to EGFR-TKIs through the repression of APAF-1, BIM, and PTEN, while miR-103 and miR-203 induce apoptosis in EGFR-TKI resistant cells, also promoting mesenchymal to epithelial transition, by downregulating PKC and SRC.

## miRNAs and new insights in overcoming resistance to EGFR-TKIs

Since miRNAs are involved in resistance to EGFR-TKIs, several studies explored the role of miRNAs in overcoming the mechanisms of resistance, with encouraging preclinical results. Zhou *et al* analysed miR-130a expression levels in H1975, A549, PC9 gefitinib-sensitive and -resistant NSCLC cell lines treated with different doses of gefitinib [75]. Interestingly, miR-130a levels were significantly lower in the gefitinib-resistant cell lines compared with the gefitinib-sensitive cell lines. Conversely, c-Met mRNA levels were higher in gefitinib-resistant than in gefitinib-sensitive cell lines. Even more importantly, it was demonstrated that transfection of miR-130a mimics could reverse resistance to gefitinib by downregulating c-Met protein levels via direct targeting of the c-Met 3’-UTR, which provides evidence for a new therapeutic strategy in reverting resistance to EGFR-TKIs [75]. Similarly, Acunzo *et al* highlighted the role of miRNAs in affecting c-Met [76]. More in details, they demonstrated that miR-130a downregulates c-Met, also reducing miR-221 and miR-222 expression. These two miRNAs have already been reported to be upregulated by c-Met activation/amplification, and are involved in cell proliferation, cell growth, and resistance to the TNF-related-apoptosis inducing ligand (TRAIL) protein in NSCLC [76–77]. Therefore, miR-130a overexpression could actually enhance apoptosis in NSCLC cells, and further studies should explore the possibility of a combined therapy with both miR-130a mimics and gefitinib, as both have been shown to revert resistance to TRAIL in human cancers [77].

More recently, Zhou *et al* also demonstrated that the forced expression of another miRNA, namely miR-34a, can inhibit cell growth and induce apoptosis in HGF-induced gefitinib-resistant HCC827 and PC-9 NSCLC cell lines via targeting c-Met [78]. Interestingly, the same study reported massive tumour regression with miR-34a plus gefitinib in mouse xenograft models of HGF-induced gefitinib-resistant tumours. The development of miR-34 as replacement therapy in EGFR-TKI resistant patients is a topic of growing interest, especially in light of its involvement in targeting c-Met and its oncogenic pathways [79]. Consistently, miR-34 has been found to inversely correlate with c-Met mRNA levels in lung, glioblastoma multiforme, and ovarian cancer [78–79]. Also, miR-34 involvement in the p53 tumour suppression pathway has been well described [80]. The importance of miR-34 in cancer development was further strengthened by Kasinski and Slack who evaluated the feasibility of the delivery of miR-34 lentivirus in order to prevent cancer initiation and progression in a therapeutically resistant mouse model of lung adenocarcinoma harbouring KRAS ^*LSL-G12D/+*^ and p53 ^*LSL-R172H/+*^ mutations [81]. Notably, miR-34 restoration induced cell-cycle arrest, apoptosis, and cellular senescence by targeting c-Met, c-myc, and the antiapoptotic protein BCL-2. These findings suggest that miR-34 replacement therapy may restore sensitivity to biological treatments in those patients in which the resistance to EGFR-TKIs is because of the amplification of MET. Moreover, miR-34, which is also able to suppress KRAS-driven cancer progression, could be of great benefit in patients whose acquired resistance depends on *KRAS* mutation. Finally, Zhao *et al* showed that miR-34 mimics enhance erlotinib sensitivity in NSCLC cell lines carrying both primary and acquired resistance to EGFR-TKIs [82], and two further studies confirmed that miR-34a might overcome resistance to EGFR-TKIs by targeting MET and AXL, which are both involved in erlotinib resistance [83–84].

More recently, Gao *et al* reported that miR-138-5 is deeply downregulated in gefitinib-resistant PC9 (harbouring delE746-A750) NSCLC cell lines, while its re-expression sensitise PC9/GR cells and another gefitinib-resistant EGFR-mutated NSCLC cell line, H1975 (harbouring both L858R/T790M mutations), to gefitinib. [85]. This phenomenon, which is mediated by G protein-coupled receptor 124, suggests that miR-138-5 replacement could be a further therapeutic approach in reversing resistance to EGFR-TKIs.

In addition, very attractive results have been obtained by Rai *et al* who reported that the injection of miR-7–expressing plasmid produces a dramatic growth arrest in either *EGFR*-mutated TKI-sensitive cell lines (PC9 and H3255) or EGFR TKI-resistant cell lines harbouring T790M mutation (R-PC9 and H1975) [86]. Importantly, no significant aberrant growth suppression was observed into A549 cells carrying wild-type EGFR and K-RAS mutations, thus highlighting that the oncogene addiction of EGFR plays a critical role in miR-7 efficacy.

As previously mentioned, EMT represents an important mechanism of acquired resistance to EGFR TKIs and different studies have shown that this process undergoes a precise regulation by specific miRNAs [54]. As a result, the possibility of reverting this process, leading to mesenchymal-to-epithelial transition through the use of miRNAs, was explored. To this regard, Lee *et al* found that miR-147 transfection in colon cancer (HCT116, SW480) and lung cancer (A-549) cell lines inhibited cell proliferation, cell migration, and reversed EMT to mesenchymal-to-epithelial transition through CDH1 upregulation and ZEB1 downregulation, thus markedly reestablishing gefitinib sensitivity [87].

Not surprisingly, also members of miRNAs biosynthetic machinery, such as the RNAse III enzyme Dicer, may be involved in TKIs resistance, as was proven by Chen *et al* who showed that Dicer expression levels are significantly higher in PC9/GR resistant cells than in PC9 cells and that Dicer knockdown restores gefitinib sensitivity in resistant cells through miR-30b/miR-30c and miR-221/miR-222 downregulation [88].

## Conclusion and perspectives

Ever since their discovery, *EGFR* mutations have represented a major step forward in the management of NSCLC patients with this genetic alteration as they were associated with exquisite sensitivity to treatment with an EGFR-TKI. However, no patient was cured, and despite initial response in most of *EGFR*-mutant patients, resistance to treatment would eventually emerge. The drugs so far developed in order to overcome such resistance, including but not limited to T790M and c-Met inhibitors have been aimed at targeting only protein-coding genes implicated in resistance to treatment. Nevertheless, we also need to explore the universe of non-coding RNAs more thoroughly. In this setting, miRNAs appear to play a crucial role in regulating lung cancer carcinogenesis, sensitivity to chemotherapy, TKIs, as well as radiotherapy [89]. Moreover, their role as diagnostic, prognostic, and predictive biomarkers has been extensively investigated and it is an emerging topic in oncology. Certainly, their clinical use in restoring sensitivity to EGFR-TKIs is corroborated by robust preclinical evidence, which is an issue that needs to be investigated further in clinical trials. In order to achieve this, we must face the challenges regarding the use of miRNAs for NSCLC treatment (e.g. difficulties in identifying the appropriate methods of miRNAs delivery, determining the optimal therapeutic regimen, confirming their safety in humans), with the ultimate goal to benefit our patients.

## Conflict of interests

The authors declare no conflict of interests.

## Figures and Tables

**Table 1.** Phase III studies in which gefitinib, erlotinib, or afatinib were compared with platinum-based chemotherapy as first-line treatment of patients with advanced NSCLC selected according to the presence of *EGFR* mutation.

	IPASS [6, 7]	First-SIGNAL [8]	NEJ002 [9, 10]	WJTOG3405 [11, 12]	OPTIMAL [13, 14]	EURTAC [15]	LUX-Lung 3 [16, 17]	LUX-Lung 6 [17, 18]
**EGFR-TKI**	Gefitinib versus carboplatin/paclitaxel	Gefitinib versus cisplatin/gemcitabine	Gefitinib versus carboplatin/paclitaxel	Gefitinib versus cisplatin/docetaxel	Erlotinib versus carboplatin/gemcitabine	Erlotinib versus platinum/based chemotherapy	Afatinib versus cisplatin/pemetrexed	Afatinib versus cisplatin/gemcitabin
***EGFR* mutation**	All	Common^∞^	All^≠^	Common^∞^	Common^∞^	Common^∞^	All	All
**Population**	Asiatic	Asiatic	Asiatic	Asiatic	Asiatic	Caucasian	Mixed	Asiatic
**RR**	71.2% versus 47.3% *P* = 0.001	84.6% versus 37.5% *P* = 0.002	74.7% versus 30.7% *P* < 0.001	62.1% versus 32.2% *P* < 0.0001	83% versus 36% *P* < 0.0001	58% versus 15% *P* < 0.05	56% versus 44% (independent) 69% versus 44% (investigator) *P* = 0.001 for both	65.7% versus 23% (independent) 74.4% versus 31.1% (investigator) *P* < 0.001 for both
**PFS**	9.5 versus 6.3 mos (HR 0.48) *P* < 0.001	8 versus 6.3 mos (HR 0.54) *P* = 0.086	10.8 versus 5.4 mos (HR 0.30) *P* < 0.001	9.2 versus 6.3 mos (HR 0.48) *P* < 0.0001	13.1 versus 4.6 mos (HR 0.16) *P* < 00001	9.7 versus 5.2 mos (HR 0.37) *P* < 0.001	11.1 vsersus 6.9 mos (HR 0.58) (all mutations, independent) 13.6 versus 6.9 mos (HR 0.47) (del19/Leu858Arg only, independent) *P* = 0.001 for both	11 versus 5.6 mos (HR 0.28) (al mutation, independent) 11 versus 5.6 mos (HR 0.25) (del19/Leu858Arg only, independent) *P* < 0.0001 for both
**OS**	21.6 versus 21.9 months, (HR 1.00) *P* = 0.990	27.2 versus 25.6 mos (HR 1.04) *P* = 0.984	27.7 versus 26.6 months (HR 0.88) *P* = 0.48	34.8 versus 37.3 months HR 1.25 *P* = NR	22.7 versus 28.9 mos (HR 1.04) *P* = 0.69	19.3 versus 19.5 mos (HR 1.04) *P* = 0.87	28.2 versus 28.2 mos (HR 0.88) *P* = 0.39	23.1 versus 23.5 mos (HR 0.93) *P* = 0.61

mos, months – NR, not reported – OS, overall survival – PFS, progression-free survival – RR, response rate

∞ del19 and L858R

≠ T790M excluded

Presented data are based on investigators’ assessment unless otherwise specified

- *EGFR*-mutation positive subgroup results; median follow-up for PFS 5.6 months [6]; median follow-up for OS 16 months [7]

- *EGFR*-mutation positive subgroup results; median follow-up for PFS and OS 35 months

- median follow-up for PFS 17.3 months [9]; median follow-up for OS 23.1 months [10]

- median follow-up for PFS 2.3 months [11]; median follow-up for OS 59.1 months [12]

- median follow-up for PFS 15.6 months [13]; median follw-up for OS 32.4 months [14]

- median follow-up not reported for the whole group of study patients

- PFS by investigator assessment for all mutations 11 versus 6.7 months, hazard ratio (HR) 0.49, *P* < 0.001; PFS by investigator assessment for del19/Leu858Arg only yielded a HR of 0.41, *P* = 0.001; median follow-up for PFS 16.4 months [16]; median follow-up for OS 41 months [17]

- PFS by investigator assessment 13.7 versus. 5.6 months, HR 0.28, *P* < 0.0001; median follow-up for PFS 16.6 months [18]; median follow-up for OS 33 months [17]

**Table 2.** Main miRNAs involved in EGFR TKIs resistance.

miRNA	Genomic location	Target genes relevant for NSCLC	Expression in TKIs resistant NSCLC	Mechanism of TKIs resistance
miR-21	17q23.2	PTEN, PDCD4, SPRY/2, APAF1, FASLG, RHOB	Upregulated	PTEN suppression PI3K/Akt pathway stimulation
miR-214	1q24.3	PTEN, CADM1	Upregulated	PTEN suppression CADM1 suppression
miR-23a/24/27a	19p13.12	CDH1	Upregulated	TGF-β1-induced EMT Loss of E-cadhein
miR-221/222	Xp11.3	TRAIL, CDKN1B, PTEN, TIMP3, PUMA, BIM, APAF1	Upregulated	Escape from TRAIL-induced apoptosis
miR-134, miR-487b, miR-655	14q32.31	MAGI2	Upregulated	TGF-β1-induced EMT Loss of PTEN function
miR-200 a/b miR-200c	1p36.33 12p13.31	ZEB1/2, FLT1, GATA3, KRAS, MAPK	Downregulated	TGF-β1-induced EMT EGFR-independent Akt activation
miR-126, miR-128b	9q34.3, 2q21.3	VEGF, CRK, SLC7A5, EGFR	Downregulated	Akt/Erk1/2 activation Angiogenesis
miR-103 a/b miR-203	5q34/20p13 14q32.33	SRC, PKC, GSK3β, AKT, ERKs	Downregulated	AKT/ERKs pathway stimulation EMT induction through SRC and PKC-ε upregulation
